# Abdominal cocoon: a rare cause of intestinal obstruction – a case report

**DOI:** 10.1093/jscr/rjag522

**Published:** 2026-06-26

**Authors:** Fatimah Y Alghareeb, Naji Fliti

**Affiliations:** Department of Surgery, Almoosa Specialist Hospital, 3256 Dhahran Road, Al Khars District, PO Box 5098, Al Mubarraz 31982, Saudi Arabia; Department of Surgery, Almoosa Specialist Hospital, 3256 Dhahran Road, Al Khars District, PO Box 5098, Al Mubarraz 31982, Saudi Arabia

**Keywords:** abdominal cocoon, sclerosing encapsulating peritonitis, small bowel obstruction, fibrous membrane

## Abstract

Abdominal cocoon, or sclerosing encapsulating peritonitis, is an uncommon cause of small bowel obstruction characterized by encasement of the intestines within a fibrous membrane. We present a case of a 75-year-old male with multiple comorbidities who presented with intestinal obstruction and was diagnosed with an abdominal cocoon intraoperatively. Preoperative diagnosis remains highly challenging due to nonspecific symptoms and clinical imaging signs. When conservative management fails, definitive surgical intervention via thorough membrane excision and careful adhesiolysis is required. Surgeons should consider abdominal cocoon as a differential diagnosis in cases of unexplained or atypical intestinal obstruction to avoid delayed management.

## Introduction

Abdominal cocoon syndrome (ACS), or idiopathic sclerosing encapsulating peritonitis, involves encasement of the small intestine within a fibrocollagenous membrane. It can be idiopathic or secondary to peritoneal dialysis, infection, or prior surgery [1, 2]. First described as “peritonitis chronica fibrosa incapsulata” in 1907 and coined “abdominal cocoon” by Foo *et al.* in 1978 [3, 4], ACS remains a diagnostic challenge preoperatively.

## Case report

A 75-year-old male with chronic obstructive pulmonary disease (COPD), hypertension, and ischemic heart disease (post-percutaneous coronary intervention) presented with eight days of abdominal pain, distension, and vomiting. He reported minimal stool passage and had failed conservative management elsewhere. On transfer, vital signs were stable (oxygen saturation 88% on room air), with marked abdominal distension and lower abdominal tenderness. Laboratory findings revealed a white blood cell count of 11.8 × 10^9^/l, hemoglobin 8.8 g/dl, and C-reactive protein 23 mg/l. Abdominal computed tomography (CT) revealed dilated small bowel loops with a transition point in the mid-ileum and a “whirling” mesenteric vessel sign (Fig. 1). Laparoscopy revealed dense adhesions, leading to conversion to open laparotomy. The small intestine was found entirely encased in a thick fibrous membrane with extensive omental adhesions (Fig. 2). Thorough adhesiolysis and complete membrane excision were performed. Postoperatively, the patient developed a transient ileus, which was managed successfully with conservative care.

**Figure 1:**
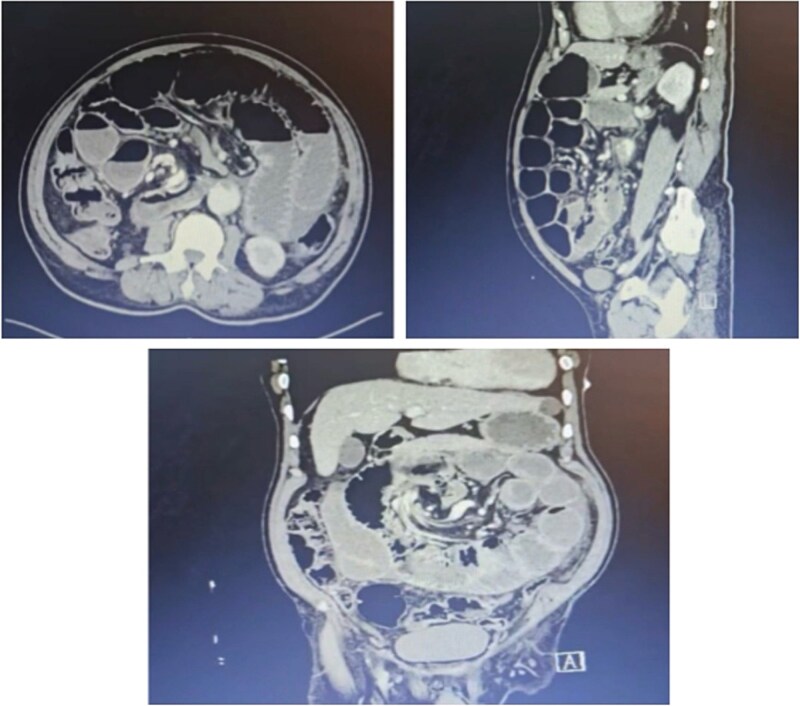
CT abdomen with IV contrast: Dilated small bowel with multiple air-fluid levels and a transition zone at the mid-ileum, showing the whirling loop sign of the mesenteric vessels, suggestive of small bowel obstruction.

**Figure 2:**
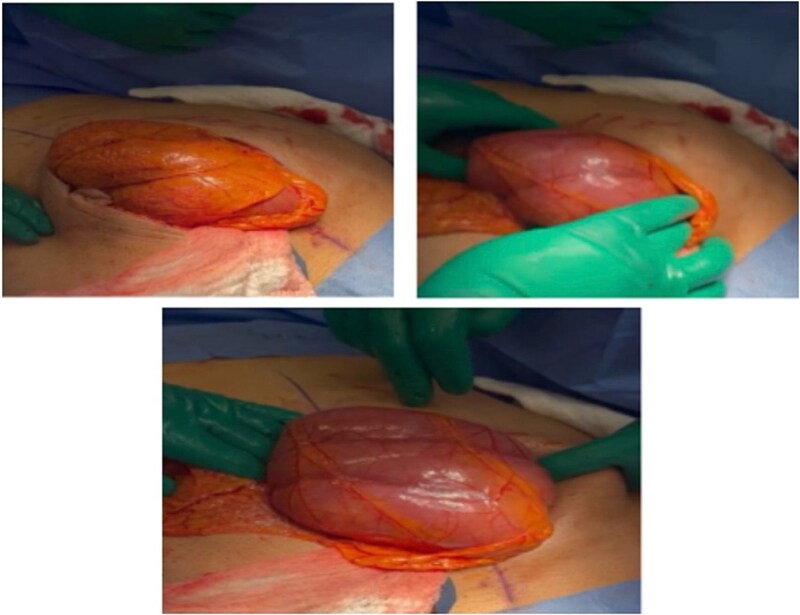
Omentum covering the small bowel, with an encapsulated membrane enveloping the small bowel (cocoon abdomen).

## Discussion

ACS is rare and typically affects young women in tropical regions, though elderly males may also be affected [5]. The etiology can be idiopathic or secondary to chronic peritoneal irritation. Clinical presentation includes intermittent obstruction, pain, and vomiting. Imaging findings such as clustered bowel loops and peritoneal thickening may suggest the diagnosis [5, 6]. Surgical intervention is indicated in complete obstruction; excision of the fibrous membrane and adhesiolysis is curative in most cases [1, 5, 6]. Our patient underwent successful surgery despite a complicated postoperative course. This case underscores the importance of considering ACS in unexplained bowel obstruction, even in atypical demographics. Early recognition and surgical management are crucial to improve outcomes.
